# Formation of Zearalenone Metabolites in Tempeh Fermentation

**DOI:** 10.3390/molecules24152697

**Published:** 2019-07-24

**Authors:** Antje Borzekowski, Riyan Anggriawan, Maryeni Auliyati, Hans-Jörg Kunte, Matthias Koch, Sascha Rohn, Petr Karlovsky, Ronald Maul

**Affiliations:** 1 Department Analytical Chemistry, Reference Materials, Bundesanstalt für Materialforschung und-prüfung (BAM), Richard-Willstätter-Str. 11, 12489 Berlin, Germany; 2 Molecular Phytopathology and Mycotoxin Research Section, University of Goettingen, Grisebachstrasse 6, 37077 Goettingen, Germany; 3 Department Materials and the Environment, Bundesanstalt für Materialforschung und-prüfung (BAM), Unter den Eichen 87, 12205 Berlin, Germany; 4 Hamburg School of Food Science, Institute of Food Chemistry, University of Hamburg, Grindelallee 117, 20146 Hamburg, Germany; 5 BfR—German Federal Institute for Risk Assessment, Max-Dohrn-Str. 8–10, 10589 Berlin, Germany

**Keywords:** modified mycotoxins, zearalenone sulfate, α-zearalenol, food fermentation, *Rhizopus*, *Aspergillus oryzae*

## Abstract

Tempeh is a common food in Indonesia, produced by fungal fermentation of soybeans using *Rhizopus sp.*, as well as *Aspergillus oryzae*, for inoculation. Analogously, for economic reasons, mixtures of maize and soybeans are used for the production of so-called tempeh-like products. For maize, a contamination with the mycoestrogen zearalenone (ZEN) has been frequently reported. ZEN is a mycotoxin which is known to be metabolized by *Rhizopus* and *Aspergillus* species. Consequently, this study focused on the ZEN transformation during tempeh fermentation. Five fungal strains of the genera *Rhizopus* and *Aspergillus*, isolated from fresh Indonesian tempeh and authentic Indonesian inocula, were utilized for tempeh manufacturing from a maize/soybean mixture (30:70) at laboratory-scale. Furthermore, comparable tempeh-like products obtained from Indonesian markets were analyzed. Results from the HPLC-MS/MS analyses show that ZEN is intensely transformed into its metabolites α-zearalenol (α-ZEL), ZEN-14-sulfate, α-ZEL-sulfate, ZEN-14-glucoside, and ZEN-16-glucoside in tempeh production. α-ZEL, being significantly more toxic than ZEN, was the main metabolite in most of the *Rhizopus* incubations, while in *Aspergillus oryzae* fermentations ZEN-14-sulfate was predominantly formed. Additionally, two of the 14 authentic samples were contaminated with ZEN, α-ZEL and ZEN-14-sulfate, and in two further samples, ZEN and α-ZEL, were determined. Consequently, tempeh fermentation of ZEN-contaminated maize/soybean mixture may lead to toxification of the food item by formation of the reductive ZEN metabolite, α-ZEL, under model as well as authentic conditions.

## 1. Introduction

Tempeh is a very common food in Indonesia. It is made of soybeans, which are fermented by molds and pressed into a compact cake. In tempeh manufacturing, predominantly molds of the genera *Rhizopus* are used; for example, *Rhizopus oryzae*, *Rhizopus oligosporus, Rhizopus microsporus,* and *Rhizopus stolonifer*, but also *Aspergillus oryzae,* were found to be in the fermenting fungal mixture [1,2]. Tempeh manufacturers in Indonesia do not use specific fungi, but inocula, which consist of a non-specified fungal mixture.

The fungal fermentation leads to an enhancement of nutritional value and digestibility, because free amino acids and other water-soluble solids are increased [3,4]. Additionally, tempeh is high in probiotics, vitamins, and minerals [5]. Therefore, it can be a nutritious addition to the diet. In Europe, the USA, and other industrialized countries, the interest for tempeh is increasing, resulting from a growing interest in health, nutrition, and vegetarianism. As soybeans contain all of the eight (or ten) essential amino acids [5], tempeh is commonly used as a vegetarian source of protein. Triggered by the high nutrient value, also, a lot of studies focused on the use of tempeh and tempeh-like products with respect to malnutrition in Third World Countries [6,7,8].

Tempeh-like products are produced out of beans other than soybeans and also cereals (e.g. wheat or maize), and cereal/soybean mixtures are used as starting material for fermentation [6,9]. For economic reasons in Indonesia, more and more tempeh-like products are on the market. Especially, tempeh-like products made out of maize/soybeans (30:70) are produced, because costs for maize are lower than for soybeans.

Contamination of the raw material with toxins, as well as a possible toxin formation during the fermentation process, is of interest with respect to food safety of a fermented product. During fermentation, there can be both toxin formation and binding, as shown in the case of malting and beer production [10,11]. In the case of tempeh, the fermentation is mainly conducted utilizing fungal strains of the genera *Rhizopus*, for which no mycotoxin formation is described. However, for some *Rhizopus* strains, endosymbiotic toxin-forming bacteria (*Burkholderia spp.*) are described, making the selection of suitable strains for industrial tempeh fermentation more demanding [12,13]. *Aspergillus oryzae*, which occasionally can be a constituent of the fungal inoculum for tempeh fermentation, can in some cases produce mycotoxins to a minor extent [14].

The maize raw material is known to be frequently contaminated with mycotoxins. Numerous studies have shown that maize can be highly contaminated, predominantly with the estrogenic *Fusarium* mycotoxin zearalenone (ZEN) [15,16,17]. ZEN is unequally distributed in different fractions of the grain. Higher ZEN concentrations were detected in by-products from cleaning, like bran and hulls, than in the clean cereal grain [18]. Adverse effects of the toxin are mediated by the hormone-like structure of ZEN, which is related to 17β-estradiol. ZEN can interact with the estrogen receptors ERα and ERβ, and can cause hormonal disorder [19]. In epidemiological studies, a chronic exposure of ZEN was associated with precocious development of children [20,21]. Especially for tempeh-like products consisting of maize and soybeans, combinatory estrogenic effects between isoflavones (present in soybeans) and ZEN may play an important role for toxicity assessment, as synergistic effects have already been shown in vitro for combinations of the phytoestrogen genistein and the mycoestrogen ZEN [22]. In addition, ZEN is immunotoxic, because it can modulate pathways of immune response and impair lymphoid organs, resulting in thymus atrophy [23].

**Figure 1:**
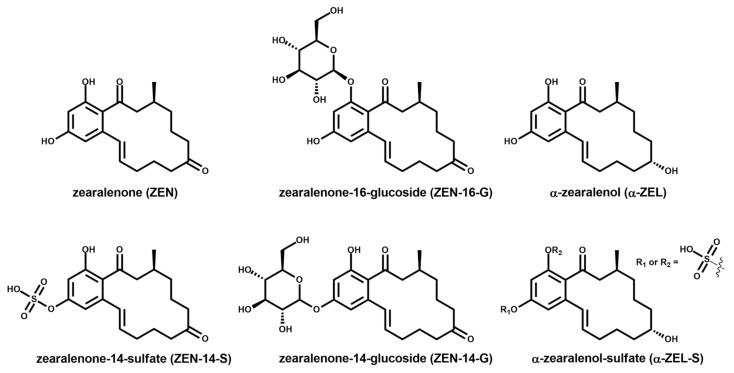
Structures of zearalenone and major zearalenone metabolites.

ZEN metabolites (Figure 1), often referred to as biologically modified forms [24,25], also play an important role for the toxicity assessment. The metabolites are formed within the detoxification process of plants and fungi, and even the ZEN-producing *Fusarium* fungus conduct sulfation of ZEN for toxicity regulation [26,27]. In general, it can be stated that plants detoxify ZEN by glucosylation, [28,29,30] and fungi such as *Aspergillus*, *Rhizopus,* or *Fusarium* species conduct sulfation and/or glucosylation of ZEN for detoxification [31,32,33]. However, sulfation has also been described as a minor detoxification route in plants [34]. After human consumption, the sulfate and glucoside conjugates of ZEN can be hydrolyzed by human intestinal microbiota [35]. Consequently, ZEN conjugates represent an additional source for human exposure to ZEN. Besides conjugated ZEN derivatives, the reductive metabolites α- and β-zearalenol (α- and β-ZEL) are formed in plant, fungal, and animal metabolism [36]. Whilst β-ZEL is less toxic than ZEN, α-ZEL possesses a higher estrogenic activity than ZEN [37]. Sulfate and glucoside derivatives of ZEL are also known to be formed in plant and fungal metabolism [31,33]. Taking the ZEN metabolites’ toxicity into account, the EU CONTAM Panel found it appropriate to set a group-based tolerable daily intake (TDI) for ZEN and its biologically modified forms [38]. The different estrogenic potentials of the ZEN derivatives were considered by the EFSA CONTAM Panel, assigning relative potency factors to the various modified forms. For the less estrogenic β-ZEL, a potency factor of 0.2 relative to ZEN was set and for α-ZEL the potency factor is 60. Moreover, for sulfate and glucoside conjugates, the same factors as for the free form are proposed.

*Rhizopus* and *Aspergillus* species used in tempeh fermentation have previously been analyzed in vivo for their capability of ZEN metabolite formation after ZEN supplementation to liquid media. All analyzed strains were able to convert ZEN to various metabolites, such as ZEN-14-sulfate (ZEN-14-S), ZEN-14- and ZEN-16-glucoside (ZEN-14-G and ZEN-16-G), α-ZEL, and α-ZEL-sulfate (α-ZEL-S) [31].

The present study focused on the investigation of the ZEN transformation during tempeh fermentation, mediated by original food technological fungal strains. In a small scale model system, applying authentic tempeh fermentation conditions, tempeh-like products were produced at a laboratory scale using fungal strains isolated from fresh Indonesian tempeh and original Indonesian inocula. The tempeh-like product manufactured out of ZEN-contaminated raw material was analyzed for transformation products of ZEN metabolites. In addition, this study comprises the analysis of tempeh-like products bought from Indonesian market, in order to conduct an initial screening for the possible occurrence of ZEN and ZEN transformation products on the market.

## 2. Results and Discussion

### 2.1. Production of Tempeh-Like Products

**Figure 2:**
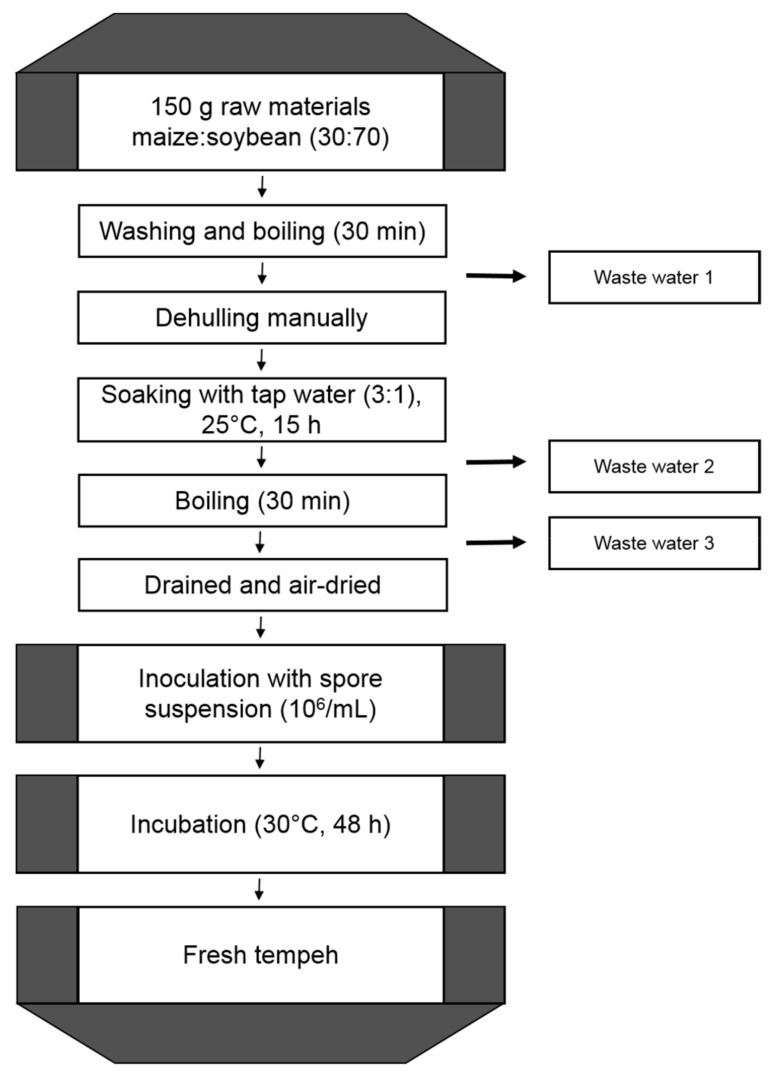
Tempeh production in laboratory-scale established based on usual Indonesian practice.

Six different procedures of tempeh manufacturing were studied in Indonesia. The comparison of the methods showed differences, especially with regard to the soaking step. Soaking during tempeh production is conducted either by natural uncontrolled acidification, controlled acidification with *Lactobacillus* strains, soaking with tempeh fungi spores, soaking with mixed yeast strains, or chemical acidification with acetic acid. In addition, one manufacturer omits boiling after soaking. In the present study, ZEN biotransformation during tempeh fermentation was investigated using the most common Indonesian method of tempeh manufacturing with natural uncontrolled acidification. The industrial method was adjusted to laboratory scale. The model procedure developed is displayed in Figure 2.

**Figure 3:**
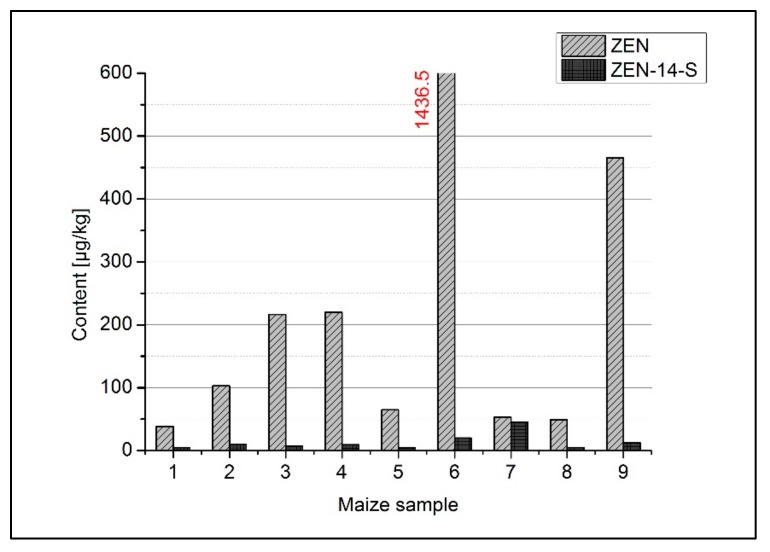
Basal contamination of maize raw material with zearalenone (ZEN) and ZEN-14-sulfate (ZEN-14-S).

The use of a maize/soybean mixture in a ratio of 30:70 as raw material was adopted from products available on the Indonesian market. For investigating ZEN metabolization during fermentation, maize naturally contaminated with ZEN was chosen. The soybeans used for blending contained neither ZEN nor ZEN derivatives. In Figure 3, the ZEN amount of the maize used in this study is shown. The determined amounts are distributed heterogeneously throughout the maize lot, ranging from 38.5 µg/kg to about 1.44 mg/kg per 12 g subsample. The large differences of the results are due to sampling. The grains were not homogenized (by milling) before usage, because the whole grain is usually used as raw material for tempeh production and very high amounts of ZEN are often located in single infected grains. Maize grains (45 g) blended with 105 g soybeans were used in each tempeh model fermentation. Therefore, the ZEN content in the maize was analyzed separately for small subsamples of grain maize, and not for a homogenous representative sample of the entire lot. A controlled spiking of raw material with ZEN would have resulted in a more homogeneous ZEN contamination. However, in order to approximate the conditions of authentic tempeh production, maize naturally contaminated with ZEN was used. Particularly, for also taking into account the washout steps in tempeh fermentation, it is more appropriate having a ZEN contamination not only on the surface of the grains (as it would have been the case for spiked grains).

Additional to the ZEN contamination, the amount of ZEN-14-S already present in the maize prior to fermentation is displayed in Figure 3, with amounts ranging from 4.55 µg/kg to 45.1 µg/kg. The concentration of each sample and the molar ratios of ZEN to ZEN-14-S are listed in Table 1. The content of the sulfated derivative formed during the metabolism of ZEN-producing *Fusarium* strains is low compared to the ZEN content, with exception of one sample (sample 7, Figure 3, Table 1), where ZEN and ZEN-14-S are present in similar amounts of 53.1 µg/kg and 45.1 µg/kg, respectively. Other sulfated or glucosylated ZEN derivatives, as well as reductive ZEN metabolites, were not detected in the maize raw material.

Furthermore, a possible leaching of toxin into the waste water was analyzed. Waste water is discarded at three points during production of the tempeh-like product (Figure 2). The average of all incubations showed an overall decrease of 27 ± 15% of ZEN for all washing, boiling, or soaking steps, where water was drained out. As ZEN is distributed unequally throughout the grains, which might also depend on the severety of the infestation, for such kernels with a superficial contamination, a more intense washout might occur. On average, about 70% of the initial ZEN remains in the fermented tempeh product in either transformed or non-modified form. The highest average ZEN concentrations were observed in waste water 1 (Figure 2), as at this step of the production process, the raw material was boiled, enhancing the solubility of ZEN and thus, the leach out into the water. ZEN losses into waste water 3, the second boiling step, were much lower in comparison to the waste water from the first boiling. In the soaking step (Figure 2, waste water 2) the loss of ZEN was negligible. The derivative ZEN-14-S was found in the waste waters in much higher relative amounts compared to ZEN, because it is more water-soluble than ZEN. When using *Rhizopus* strains for fermentation of the tempeh-like product, the amounts of ZEN-14-S in the waste water were up to 10-times higher compared to the final tempeh, leading to the conclusion that most of the ZEN-14-S is leached out. By contrast, the amount of ZEN-14-S in the final tempeh-like product was higher than the total amount in the waste water when using *Aspergillus oryzae* for the incubations. This result indicates that ZEN-14-S is formed out of ZEN during fermentation with *Aspergillus oryzae*.

ZEN metabolization during fermentation of the tempeh-like product was investigated by the use of five authentic fungal strains: *Rhizopus oryzae, Rhizopus microsporus* var. *chinensis, Aspergillus oryzae* and two different *Rhizopus microsporus* var. *oligosporus* strains (Table 2). These strains, isolated from fresh Indonesian tempeh and authentic Indonesian inocula, represent the common fungi which are present in undefined fermentation inocula of industrial tempeh production. The strains were assigned to species based on morphological characters (sporangium, sporangiospore, columellae, zygospore, rhizoid type and colony appearance). The assignment was confirmed by sequencing the actin gene.

### 2.2. Zearalenone Metabolization during Fermentation of Tempeh-Like Products

**Figure 4:**
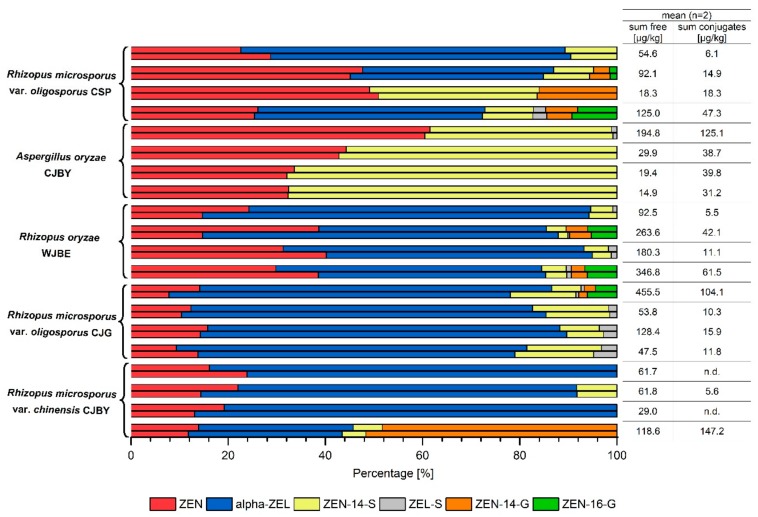
Content and relative distribution of zearalenone (ZEN), α-zearalenol (α-ZEL), ZEN-14-sulfate (ZEN-14-S), α-ZEL-sulfate (ZEL-S), ZEN-14-glucoside (ZEN-14-G), and ZEN-16-glucoside (ZEN-16-G) in tempeh-like products fermented with the strains *Rhizopus microsporus* var. *oligosporus* CSP, *Aspergillus oryzae* CJBY, *Rhizopus oryzae* WJBE, *Rhizopus microsporus* var. *oligosporus* CJG, *Rhizopus microsporus* var. *chinensis* CJBY; for each fungal strain, fermentation was conducted in quadruplicate; each incubation was analyzed twice; sum free includes ZEN and α-ZEL amount; sum conjugates includes amount of the analyzed ZEN and ZEL conjugates.

Separate fermentations were conducted four times for each of the five fungal strains in portions of 150 g maize/soya mixture. After fermentation the samples were freeze-dried and homogenized by grinding. Every fermented mixture was analyzed in duplicate for the occurrence of ZEN metabolites. Whilst the initial soybeans were free of mycotoxins and in the maize ZEN and relatively low amounts of ZEN-14-S were present, the analyses showed for all fermented samples higher amounts and a diverse pattern of conjugates and reductive metabolites. The formation of α-ZEL, ZEN-14-S, ZEN-14-G, ZEN-16-G and α-ZEL-S was observed (Figure 4). However, β-ZEL and its sulfate as well as ZEL-glucosides were not detected in the fermented tempeh-like product. This result is in line with previous results from liquid culture experiments [31] and expecially the absence of β-ZEL shows that the metabolism of the investigated fungi is significantly different from other species such as mammals or insects always forming α-ZEL along with β-ZEL [36,39]. Moreover, all tempeh-like products were also screened for presence of diglucosidic and disulfated derivatives. However, none of these derivatives were found.

General differences were observed between the fermentations with *Aspergillus oryzae* or *Rhizopus* species. The analyzed *Rhizopus* strains metabolized ZEN to α-ZEL and glucosidic and sulfated conjugates, whereas *Aspergillus oryzae* only formed sulfate metabolites. These results confirm previous in vivo investigations on the biotransformation of ZEN by *Rhizopus* and *Aspergillus* species under food technological conditions [31].

In contrast to the in vivo analyses described by Brodehl et al. [31] where conjugated ZEN derivatives were the main metabolites, now α-ZEL represents the main metabolite in most of the *Rhizopus* incubations. Nevertheless, differences in metabolite formation can be observed between the different *Rhizopus* strains. *Rhizopus microsporus* var. *chinensis* CJBY formed α-ZEL exclusively in two out of the four incubation replicates. In the other two incubations, 6 µg/kg and 15 µg/kg of ZEN-14-S were determined additionally to α-ZEL. These relatively low amounts of ZEN-14-S were probably originating from the maize used for fermentation, which in some cases was already initially contaminated with ZEN-14-S (Figure 3, Table 1).

In one of the *Rhizopus microsporus* var. *chinensis* CJBY incubations, 50% of the initial ZEN was metabolized to ZEN-14-G. This relatively high glucoside formation is hypothesized to result from the very high initial ZEN content in the maize of about 700 µg/kg. For calculation of the ZEN contamination prior to fermentation, an exclusive conversion of ZEN to the analyzed metabolites was assumed and calculated on a molar basis. Formation of ZEN-14-G and ZEN-16-G additional to sulfate conjugates and α-ZEL formation was also observed for *Rhizopus microsporus* var. *oligosporus* CJG and *Rhizopus oryzae* WJBE when the initial ZEN contamination of the maize was high. This leads to the assumption that the *Rhizopus* fungi are capable of catalyzing glucosylation as an additional detoxification process at elevated toxin levels. However, in one incubation *of Rhizopus microsporus* var. *oligosporus* CSP, the initial ZEN amount was relatively low and ZEN-14-G and ZEN-14-S were formed, as well.

α-ZEL-S formation was observed as a minor metabolite in some incubations with *Rhizopus sp.* and one *Aspergillus oryzae* incubation. The main sulfated conjugate was ZEN-14-S, detected particularly in *Aspergillus oryzae* incubations with amounts of 31.2 µg/kg to 122.1 µg/kg. The ZEN-14-S formation in all incubations cannot be assigned unambiguously to the fermentation process, because of the unknown amount of ZEN-14-S in the raw material. Taking into account the detected amounts of ≤ 45.1 µg/kg of ZEN-14-S in raw maize samples (Figure 3, Table 1), together with the dilution resulting from the blending with mycotoxin-free soya, a high probability of additional ZEN-14-S formed by fermentation with *Aspergillus oryzae* can be assumed. This assumption becomes even more likely as before fermentation, significant losses of initial ZEN-14-S from the raw material occur due to washing, boiling, and soaking during tempeh production.

The molar ratios of ZEN to modified forms in Table 3 show that most of the tempeh-like model samples contain more ZEN derivatives than ZEN as a result of the fungal fermentation process. In comparison to Table 1, where molar ratios of ZEN to ZEN-14-S in the raw material of >1.0 are given, a change in the molar ratio of ZEN to modified forms to a quotient of less than 1.0 in almost all cases was observed.

### 2.3. Analysis of Authentic Tempeh-Like Products

**Figure 5:**
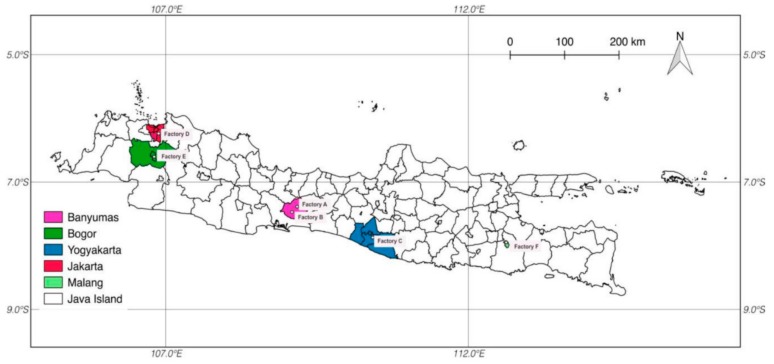
Sampling map (Java, Indonesia).

Authentic tempeh-like product samples, all produced from a maize/soybean mixture, were collected from Indonesian markets. In total, 14 tempeh products from three Indonesian regions (East Java, Central Java, and West Java, see Figure 5) were analyzed for the occurrence of ZEN and ZEN metabolites. Two tempehs were contaminated with ZEN, α-ZEL and ZEN-14-S, and two tempehs were contaminated with ZEN and α-ZEL (Table 4). These findings support the results obtained for the tempeh model fermentations. However, in the authentic tempeh-like product samples, the amounts of α-ZEL were exceeding the ZEN amounts. The distribution of ZEN and α-ZEL in untreated grain samples was significantly different. In maize and other grains, the content of ZEN is usually much higher than the α-ZEL content, or even no α-ZEL is detected additional to ZEN [16,40]. Therefore, higher amounts of α-ZEL compared to ZEN indicate that α-ZEL was formed during the original tempeh fermentation.

The formation of glucosylated ZEN derivatives or α-ZEL-S was not observed in the authentic samples. The metabolization processes vary strongly depending on the genera and the composition of the fermenting fungi in the inocula. Neither the fungal composition of the inocula nor the contained fungi were known. Thus, the formation of ZEN metabolites cannot be compared directly to any of the model fermentations. Nevertheless, *Rhizopus* were the dominant species in the mixtures, and the formation of α-ZEL as the main metabolite was observed in the model system, as well as in the authentic samples.

## 3. Materials and Methods

### 3.1. Chemicals and Media

Potato dextrose agar (PDA) and potato dextrose broth (PDB) were prepared using instant media purchased from Carl Roth GmbH & Co (Karlsruhe, Germany). The soya for tempeh fermentation was bought from Indonesian market. Dr. Christine Schwake Anduschus (Max Rubner-Institut, Federal Research Institute of Nutrition and Food, Detmold, Germany) kindly provided the ZEN-contaminated maize. The uncontaminated maize as the negative control was purchased in a local market, produced by Herbert Kluth GmbH & Co. KG (Henstedt-Ulzburg, Germany). ZEN was acquired from Bio-Techne GmbH (Wiesbaden, Germany). α-ZEL and β-ZEL were purchased from Sigma-Aldrich GmbH (Steinheim, Germany). A certified Biopure solution of U-[^13^C_18_]-ZEN (25.1 ± 0.7 µg/mL) was obtained from Romer Labs Austria (Tulln, Austria). ZEN-14-G, ZEN-14-S, and ZEN-16-G were synthesized according to Borzekowski et al. [27]. Ammonium acetate was purchased from Mallinckrodt Baker Inc. (Griesheim, Germany). Acetonitrile was of HPLC-grade and was obtained from Th. Geyer GmbH & Co. KG (Renningen, Germany). Ultrapure water was obtained from a Seralpur PRO 90 CN purification system by Seral Reinstwasser GmbH (Ransbach-Baumbach, Germany).

### 3.2. Fungal Strains and Growth Conditions

*Rhizopus microsporus* var. *oligosporus* CSP, *Aspergillus oryzae* CJBY, *Rhizopus oryzae* WJBE, *Rhizopus microsporus* var. *oligosporus* CJG, and *Rhizopus microsporus* var. *chinensis* CJBY isolated from fresh Indonesian tempeh and authentic inocula were used for tempeh fermentation. Stock cultures were grown on potato dextrose agar (PDA) media for six days at 30 °C. The spore suspension for the inoculation in tempeh production was prepared by diluting mycelium of the stock culture in sterile water. The spore suspension was determined by counting in Thoma-chamber (0.1 mm depth, 0.0025 mm^2^) under the light microscope (Carl Zeiss AG, Oberkochen, Germany) with 10x magnifications. The final densities of spore suspension were adjusted to 10^6^ spores/mL.

### 3.3. Isolation and Identification of Fungal Strains

The strains were assigned to species based on morphological characters (sporangium, sporangiospore, columellae, zygospore, rhizoid type, and colony appearance) according to Schipper et al. [41], Liou et al. [42], and Zheng et al. [43], and carbon assimilation profiles were recorded with the ID32C system (bioMérieux SA, Marcy-l’Etoile, France). The assignment was confirmed by sequencing the actin gene [44].

### 3.4. Production of Tempeh-Like Products in Laboratory Scale

For every tempeh sample, a mixture of 105 g soybeans and 45 g maize was prepared and then put in a 1.5 L flask. The raw materials were washed and boiled for 30 min in tap water. After boiling, the water was discarded, and some was collected for sampling (waste water 1). Soybeans were dehulled manually. Dehulled raw materials were soaked in 450 mL tap water for 15 h at 25 °C and then the water was discarded, and some was collected as sample (waste water 2). The materials were boiled again in tap water for 30 min and then the boiling water was discarded, and some was collected as the waste water 3 sample. Tempeh materials were drained and air-dried at room temperature. Spore suspension (7.5 mL) with spore density 10^6^ spores/mL was added to the materials. Materials and inoculum were mixed thoroughly and then placed in petri dishes. Tempeh was incubated for 48 h at 30 °C for the fermentation.

### 3.5. Analysis of Tempeh Raw Material, Waste Water, and Tempeh-Like Products

Initially, analysis of tempeh raw material (maize and soybeans) was conducted on the whole seeds. Whole grain (10 g) of maize or soybeans were milled and 2 g of the flour were extracted with 20 mL acetonitrile/water (80:20 v/v) for 3 h at a horizontal shaker HS 501 digital (IKA^®^-Werke GmbH & Co. KG, Staufen, Germany). The supernatant was used directly for HPLC-MS/MS (high-performance liquid chromatography tandem mass spectrometry) analysis. A 9-fold determination was carried out for each batch, to take into account sample inhomogeneity of the whole grain batch.

Analysis of the content of ZEN and ZEN derivatives in the drained waste water was carried out by addition of 500 µL ice-cold acetonitrile (for protein precipitation) to 500 µL waste water. The samples were stored overnight at 4 °C and centrifuged at 11 500 g at room temperature for 5 min. The supernatant was transferred into a HPLC vial and analyzed with HPLC-MS/MS.

Analysis of the amount of ZEN and ZEN derivatives in tempeh-like products was carried out after freeze-drying and milling. Tempeh flour (2.5 g) was extracted with 20 mL acetonitrile/water (80:20 v/v) for 3 h with 1/300 min at a horizontal shaker HS 501 digital (IKA^®^-Werke GmbH & Co. KG, Staufen, Germany). The supernatant was used for direct analysis by HPLC-MS/MS.

First, all extracts were screened for the presence of ZEN, ZEL, ZEN-S, ZEN-G, ZEL-S, ZEL-G, and in addition, for diglucosylated and disulfated ZEN and ZEL derivatives. Afterwards for ZEN conjugates present, matrix matched calibrations were carried out for quantification. ZEN was quantified using [^13^C_18_]-ZEN as internal standard. The internal standard [^13^C_18_]-ZEN was added to the tempeh or flour sample before extraction or directly to the waste water before addition of acetonitrile.

### 3.6. HPLC-MS/MS Analysis

HPLC-MS/MS (high-performance liquid chromatography tandem mass spectrometry) analysis was performed on a 1100 series HPLC system from Agilent Technologies Deutschland GmbH (Waldbronn, Germany) connected to an API 4000 triple-quadrupole MS/MS system from SCIEX (Framingham, MA, USA). The analytical column was a Synergi Polar-RP (150 mm × 3.0 mm, particle size 4 µm, pore size 80 Å) in combination with a corresponding guard column (Phenomenex Ltd., Aschaffenburg, Germany). The column temperature was set to 30 °C. Solvent A was water with 5 mM ammonium acetate and solvent B acetonitrile/water (99:1 v/v) with 5 mM ammonium acetate. The gradient used was composed as follows: 0–2 min isocratic with 10% B, 2–4 min linear to 40% B, 4–10 min linear to 100% B, isocratic 10–13 min 100% B, shifting back to 10% B and reconditioning from 13–17 min. The flow rate of the mobile phase was 0.7 mL/min and 10 µL was used as standard injection volume. The ESI interface was operated in negative ionization mode at 450 °C with the following settings: Curtain gas 20 psi, nebulizer gas 60 psi, heater gas 60 psi, ionization voltage −4500 V. MS/MS measurements were exclusively conducted in selected reaction monitoring (SRM) mode. Two mass transitions were recorded for each analyte with *m/z* ratios corresponding to [M-H]^−^ ionization: ZEN *m/z* 317.0→130.8 (declustering potential (DP) −15 V, collision energy (CE) −40 eV), *m/z* 317.0→174.8 (DP = −15 V, CE = −30 eV); ^13^C_18_-ZEN *m/z* 335.2→140.2 (DP = −80 V, CE = −40 eV); ZEN-sulfate *m/z* 397.1→317.1 (DP = −65 V, CE = −30 eV), *m/z* 397.1→175.0 (DP = −65 V, CE = −50 eV); ZEN-glucoside *m/z* 479.1→317.0 (DP = −65 V, CE = −16 eV), *m/z* 479.1→130.8 (DP = −65 V, CE = −50 eV); ZEL *m/z* 319.2→174.0 (DP = −30 V, CE = −30 eV), *m/z* 319.2→160.0 (DP = −75 V, CE = −30 eV); ZEL-sulfate *m/z* 399.2→319.2 (DP = −30 V, CE = −30 eV), *m/z* 399.2→275.2 (DP = −30 V, CE = −40 eV); ZEL-glucoside *m/z* 481.2→319.2 (DP = −65 V, CE = −16 eV), *m/z* 481.2→275.2 (DP = −65 V, CE = −30 eV); ZEN-diglucoside *m/z* 641.3→317.1 (DP = −30 V, CE = −30 eV), *m/z* 641.3→479.1 (DP = −30 V, CE = −30 eV); ZEN-disulfate *m/z* 477.1→317.1 (DP = −30 V, CE = −30 eV), *m/z* 477.1→397.1 (DP = −30 V, CE = −30 eV). For diglucosylated and disulfated ZEN derivatives, ions with *m/z* ratios corresponding to [M-H]^−^ of the assumed conjugates were allowed to pass the first quadrupole (Q1) for fragmentation in Q2. Q3 was set to the *m/z* values of deprotonated ZEN (*m/z* 317.1) or the deprotonated monoconjugated form. Default values were used to monitor diglucosylated and disulfated ZEN derivatives analogous to Berthiller et al. [29]. For screening, a signal-to-noise ratio of 3:1 was applied as limit of detection (LOD). Limit of quantification (LOQ) for each analyte was at minimum a signal-to-noise ratio of 10:1. On the basis of the matrix-matched calibration level LOD and LOQ were estimated in tempeh extracts, as tempeh is the most complex matrix: LOD (ZEN) = 1.3 µg/kg; LOQ (ZEN) = 4.3 µg/kg; LOD (α-ZEL) = 5.4 µg/kg; LOQ (α-ZEL) = 18.1 µg/kg; LOD (ZEN-14-S) = 1.5 µg/kg; LOQ (ZEN-14-S) = 5.0 µg/kg; LOD (ZEN-14-G) = 1.4 µg/kg; LOQ (ZEN-14-G) = 4.5 µg/kg; LOD (ZEN-16-G) = 1.0 µg/kg; LOQ (ZEN-16-G) = 3.4 µg/kg.

### 3.7. Semi-Quantification of α-ZEL-S

Semi-quantitative measurements were conducted for α-ZEL-S by using a relative response factor of α-ZEL-S to α-ZEL of 16. The response factor was estimated by comparing the MS/MS peak area before and after quantitative sulfate cleavage. The enzymatic hydrolysis of the sulfate ester was carried out according to Brodehl et al. [31].

## 4. Conclusions

This study showed for the first time, that ZEN is transformed to its metabolites by fungal fermentation during production of tempeh-like products. In contrast to previous in vivo findings [29], the formation of ZEN conjugates played a less important role. The main metabolite in most of the *Rhizopus* fermentations in the present study was α-ZEL, which was also observed for contaminated authentic samples from Indonesia, which dominantly contained fungi of the genera *Rhizopus* with α-ZEL as the main metabolite. Taking into account that there are no legal limits in Indonesia for ZEN in maize, highly contaminated maize might be used for production of tempeh-like products. As a result, transformation of ZEN to the more estrogenic metabolite α-ZEL is a potential health risk for the consumers of especially tempeh-like products, because the estrogenic potency of α-ZEL compared to ZEN is up to 60-fold higher [38]. Thus, the use of ZEN-contaminated maize may result in an increase of a health risk, due to fermentation and accompanying processes transforming ZEN into α-ZEL.

Moreover, tempeh-like products represent a potential additional source of human exposure for the modified forms of ZEN and ZEL, as the investigated fungi catalyze the formation of glucoside and sulfate conjugates. As validated methods for the conjugates are still missing, this additional exposure is prone to remaining undetected. To decrease the toxin amount of the raw material, further studies can focus on additional washing steps, because the investigations in the present study also showed that the initial contaminations with ZEN and ZEN-14-S were partly washed out. In addition, an overview of the contamination of tempeh-like products with mycotoxins in general, including their metabolites, are of certain interest, because cereals are often contaminated with further mycotoxins such as aflatoxins [45]. Due to the very common combined use of maize and soy beans for tempeh-like products, the combinatory effects of the phytoestrogenic soy isoflavones and the mycoestrogen ZEN should be evaluated for this product in future.

## Figures and Tables

**Table 1:** Content and molar ratio of zearalenone (ZEN) and ZEN-14-sulfate (ZEN-14-S) in maize raw material.

Maize Sample	Content (µg/kg)		Molar Ratio ZEN/ZEN-14-S
	ZEN	ZEN-14-S	
1	38.5	5.09	9.5
2	102.9	10.3	12.6
3	216.4	7.18	37.7
4	220.1	9.43	29.2
5	65.1	4.55	17.9
6	1436	20.1	89.4
7	53.1	45.1	1.5
8	49.6	4.86	12.8
9	465.7	12.2	47.7

**Table 2:** Origin of fungal strains used for tempeh fermentation.

Tempeh Fungi	Strain	Origin
*Rhizopus oryzae*	WJBE7.84	West Java
*Rhizopus microsporus* var. *chinensis*	CJBY16.192	Central Java
*Aspergillus oryzae*	CJBY22.260	Central Java
*Rhizopus microsporus* var. *oligosporus*	CJG27.324	Central Java
*Rhizopus microsporus* var. *oligosporus*	CSP71.850	Central Sulawesi

**Table 3:** Molar ratio of zearalenone (ZEN) and biologically modified ZEN in tempeh-like products fermented with the strains *Rhizopus microsporus* var. *oligosporus* CSP, *Aspergillus oryzae* CJBY, *Rhizopus oryzae* WJBE, *Rhizopus microsporus* var. *oligosporus* CJG and *Rhizopus microsporus* var. *chinensis* CJBY; four replicates for each strain as indicated by appendices (a) to (d).

Tempeh-Like Product	Molar Ratio ZEN/Modified ZEN
*Rhizopus microsporus* var. *oligosporus* CSP (a)	0.4
*Rhizopus microsporus* var. *oligosporus* CSP (b)	0.9
*Rhizopus microsporus* var. *oligosporus* CSP (c)	1.4
*Rhizopus microsporus* var. *oligosporus CSP* (d)	0.4
*Aspergillus oryzae CJBY* (a)	1.9
*Aspergillus oryzae CJBY* (b)	0.9
*Aspergillus oryzae* CJBY (c)	0.6
*Aspergillus oryzae* CJBY (d)	0.6
*Rhizopus oryzae* WJBE (a)	0.2
*Rhizopus oryzae WJBE* (b)	0.2
*Rhizopus oryzae* WJBE (c)	0.7
*Rhizopus oryzae* WJBE (d)	0.7
*Rhizopus microsporus* var. *oligosporus* CJG (a)	0.1
*Rhizopus microsporus* var. *oligosporus* CJG (b)	0.1
*Rhizopus microsporus* var. *oligosporus* CJG (c)	0.2
*Rhizopus microsporus* var. *oligosporus* CJG (d)	0.2
*Rhizopus microsporus* var. *chinensis* CJBY (a)	0.3
*Rhizopus microsporus* var. *chinensis* CJBY (b)	0.2
*Rhizopus microsporus* var. *chinensis* CJBY (c)	0.2
Rhizopus microsporus var. chinensis CJBY (d)	0.2

**Table 4:** Contamination of authentic tempeh-like product samples with ZEN and ZEN metabolites.

Sample	Origin	Content (µg/kg)						
		ZEN	α-ZEL	β-ZEL	ZEN-14-S	α-ZEL-S	ZEN-14-G	ZEN-16-G
**TY2**	Yogyakarta, Central Java	-	-	-	-	-	-	-
**TY4**	Yogyakarta, Central Java	-	-	-	-	-	-	-
**TB2**	Banyumas, Central Java	-	-	-	-	-	-	-
**TB3**	Banyumas, Central Java	-	-	-	-	-	-	-
**TB4**	Banyumas, Central Java	17.50	33.99	-	-	-	-	-
**TM3**	Malang, East Java	9.08	43.63	-	16.31	-	-	-
**TM4**	Malang, East Java	-	-	-	-	-	-	-
**TM5**	Malang, East Java	-	-	-	-	-	-	-
**TM6**	Malang, East Java	24.75	28.54	-	15.75	-	-	-
**TJ1**	Jakarta, West Java	-	-	-	-	-	-	-
**TJ2**	Jakarta, West Java	-	-	-	-	-	-	-
**TJ3**	Jakarta, West Java	-	-	-	-	-	-	-
**TBG1**	Bogor, West Java	-	-	-	-	-	-	-
**TBG3**	Bogor, West Java	8.34	28.08	-	-	-	-	-

“-“ corresponds to < LOQ.
